# Augmented Reality in Healthcare

**DOI:** 10.1155/2019/9321535

**Published:** 2019-07-01

**Authors:** Vincenzo Ferrari, Gudrun Klinker, Fabrizio Cutolo

**Affiliations:** ^1^Dipartimento di Ingegneria dell'Informazione, Università di Pisa, Largo Lucio Lazzarino, 2, 56122 Pisa, Italy; ^2^Institut für Informatik, München Technische Universität München, Boltzmannstraße 3 D-85748 Garching b. München, Munich, Germany

Augmented reality (AR) technologies for the consumer market are nowadays mature for many potential fields of applications. In the healthcare sector, as demonstrated by the increasing number of publications on AR for surgery, medicine, and rehabilitation, there is a great demand for solutions that are able to improve current clinical practice. The aim of this special issue is to offer to engineers, computer scientists, and final users an overview of the potentials of AR technologies in fostering the development of useful applications in the early future and to steer the academic research towards overcoming the technological and human-factor issues still present among the current devices and among the most popular modalities for enriching the visual sensation with computer-generated elements.

Sixteen papers were submitted for this special issue. Our distinguished reviewers from respective research fields narrowed the field down to six papers which were finally accepted.

In this special issue, the reader can find useful examples of applications in the healthcare domain from doctor-patient communication up to surgery, rehabilitation, and phobia treatments.

Even if AR devices and applications are to date mostly devoted to augmenting the sense of sight, and the augmentation of different senses has not yet reached the same widespread diffusion, Z. Qin et al. show us in their work the potential of haptic feedback towards increasing the user's accessibility and allowing an intuitive and natural interaction with computer-generated elements.

From a technological standpoint, it is important to outline that, as confirmed in R. Touati et al., video-based tracking can be done through feature detection on the patient with a marker-less tracking approach.

Overall, it is often difficult to decide where exactly within the reality-virtuality continuum a specific AR application should be located. This is especially true for medical AR, where a lot of patient-specific data and images are available and sometimes it is almost impossible to clearly define to what extent a digital content shown on a display is real or virtual. In some research works in the healthcare sector, this debate becomes a pure comparison between VR and AR while the final goal of the application is lost. In this special issue, the reader can see that there are many ways by which the real and virtual information can be acquired and merged in a useful way for the user. M. Melero et al. show that, for some applications, the visualization of both VR and AR modalities can be an added value for the patient, while C.-F. Tsai et al. prove that the VR and AR visualization modalities stimulate different physiological reactions.

In almost all proposed applications, the AR view is shown on a traditional stand-up monitor. In the case of endoscopic procedures, as in V. Mamone et al., this choice is the best in terms of ergonomics since the users usually see the endoscope images directly in front of them. In principle, in the case of manual procedures, the optimal choice should be the use of a wearable display but, as confirmed by S. Condino et al., there are still perceptual limits to take into account, such as a parallax error and/or a focus rivalry between real and virtual, that can compromise their efficacy in a real scenario.

